# Collision tumor with inflammatory breast carcinoma and malignant phyllodes tumor: a case report and literature review

**DOI:** 10.1186/1477-7819-12-5

**Published:** 2014-01-08

**Authors:** Young Duck Shin, Seul Kee Lee, Kyu Sun Kim, Mi Ja Park, Joo Heon Kim, Hyun Sun Yim, Young Jin Choi

**Affiliations:** 1 Department of Anesthesiology, Chungbuk National University School of Medicine, Cheongju 361-711, Korea; 2 Department of Surgery, Eulji University Hospital, Eulji University School of Medicine, Dunsan-dong 1306, Seo-gu, Daejeon 135-710, Korea; 3 Department of Radiology, Eulji University Hospital, Eulji University School of Medicine, Dunsan-dong 1306, Seo-gu, Daejeon 135-710, Korea; 4 Department of Pathology, Eulji University Hospital, Eulji University School of Medicine, Dunsan-dong 1306, Seo-gu, Daejeon 135-710, Korea

**Keywords:** Breast, Neoplasm, Phyllodes tumor, Ductal carcinoma, Inflammatory breast carcinoma

## Abstract

There have been some reports of coincidental presentation of breast carcinoma and phyllodes tumor in the same breast. Most of the cases were carcinoma that arose from a phyllodes tumor with a histologically identified transitional area, and they behaved less aggressively than the usually encountered carcinoma. Collision tumors are rare clinical entities in which two histologically distinct tumor types show involvement at the same site. The occurrence of these tumors in the breast is extremely rare. Here, we report a case of 45-year-old woman who had both invasive ductal carcinoma as the finding of inflammatory carcinoma and a malignant phyllodes tumor in the same breast. There was no evidence of a transitional area between the phyllodes tumor and the invasive ductal carcinoma. To our knowledge, this is the first report of a collision tumor of inflammatory breast carcinoma coincident with a malignant phyllodes tumor in same breast.

## Background

Collision tumors are rare clinical entities in which two histologically distinct tumor types show involvement in the same site. Coincidental occurrence of breast carcinoma and axillary lymphoma has been reported with a variant subtype of lymphoma and leukemia [1,2]. But, the presentation of carcinoma and other tumors in same breast as a finding of collision tumor is extremely rare, and there have been few reports of collision tumor consisting of invasive ductal carcinoma admixed with breast mucosa-associated lymphoid tissue lymphoma [3], chronic lymphocytic leukemia and lactating adenoma [4]. The epithelial component of phyllodes tumors (PTs) can transform to carcinoma and the type of carcinoma that has been reported varied and include *in situ* carcinoma invasive ductal, tubular, lobular and squamous carcinoma types [5-7]. These lesions reported in the literature showed a histologically identified transitional area, and they behaved less aggressively than the usually encountered carcinoma. The present case of collision tumor, which consisted of PT and invasive carcinoma in the same breast, is extremely rare;in a review of the literature, we found three cases of collision tumor with malignant PT and invasive carcinoma [8-10].

Here, we report a case of a 45-year-old woman who had both malignant PT with invasive carcinoma as the finding of inflammatory breast carcinoma with massive lymph node (LN) involvement. There was no evidence of a definite transition between two types of tumor, the patient was diagnosed as having a collision tumor with carcinoma and malignant PT. To our knowledge, this is the first report of a collision tumor of the breast that consisted of inflammatory carcinoma and malignant PT.

## Case presentation

**Figure 1:**
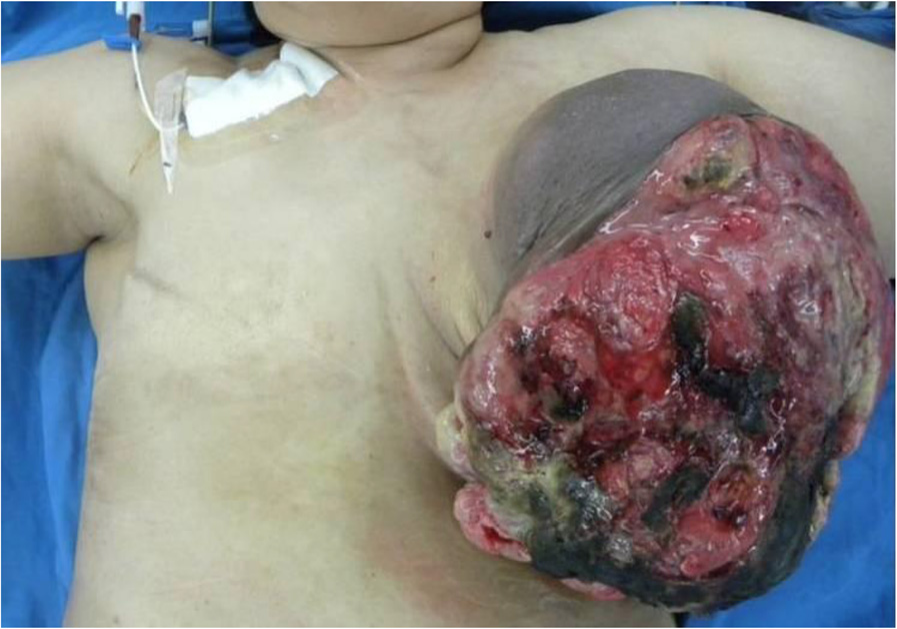
Bulky tumor mass with extensive necrosis and ulceration was seen on left breast.

A 45-year old woman presented with a huge left breast mass that had necrosis and a foul odor. She had a history of contralateral metaplastic carcinoma, and she had undergone a right modified radical mastectomy 15 years previously. She also presented with a 2-cm-sized painless palpable breast mass in her left breast two years before presentation for the case in this report, but she did not undergo any clinical work-up at that time. Then, approximately 2 or 3 months prior to presentation of this case, she noticed a mass extrusion of the outside skin with rapid growth. On physical examination, a huge necrotic mass 20 cm in diameter was seen in the entire left breast. The mass was extruded skin outside, and skin overlying the mass was thickened with edematous change (Figure 1). The axilla showed multiple conglomerated lymph nodes (LNs) that were fixed to the chest wall.

**Figure 2:**
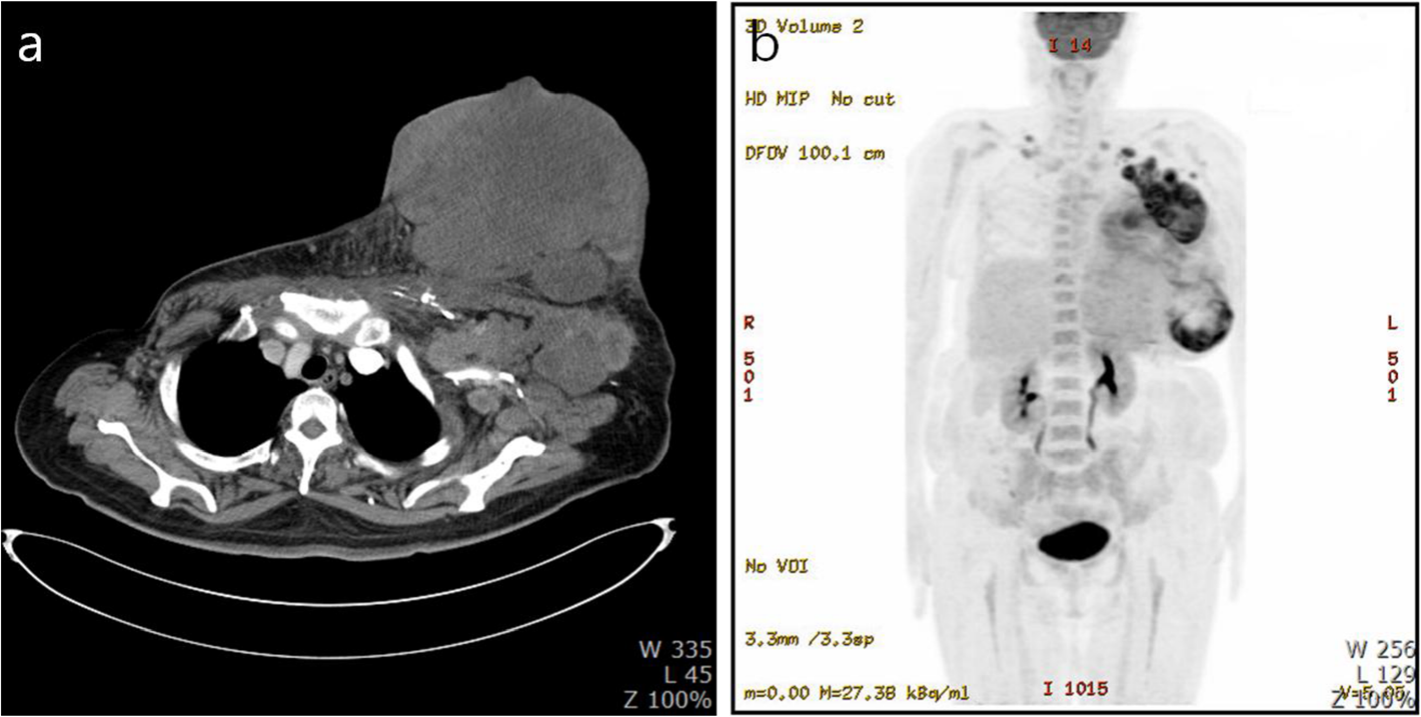
**Image findings. (a)** Enhanced chest computed tomography (CT) showing a huge breast mass with necrosis and multiple lymph node (LN) enlargements in the left axilla, both supraclavicular area. **(b)** 18 F-FDG positron emission tomography (PET)/CT revealed a huge uneven hypermetabolic mass and massive hypermetabolic LN metastases without the evidence of distant organ metastasis.

A chest computerized tomography (CT) showed a 22-cm-sized huge breast mass with necrotic changes and multiple LN enlargements in the left axilla, both supraclavicular and parasternal areas including the left internal mammary and paracardiac LNs (Figure 2a). On ^18^ F-FDG positron emission tomography (PET)/CT, a huge uneven hypermetabolic mass was seen in the left breast, and there was no evidence of distant organ metastasis except for extensive hypermetabolic LN metastasis (Figure 2b).

**Figure 3:**
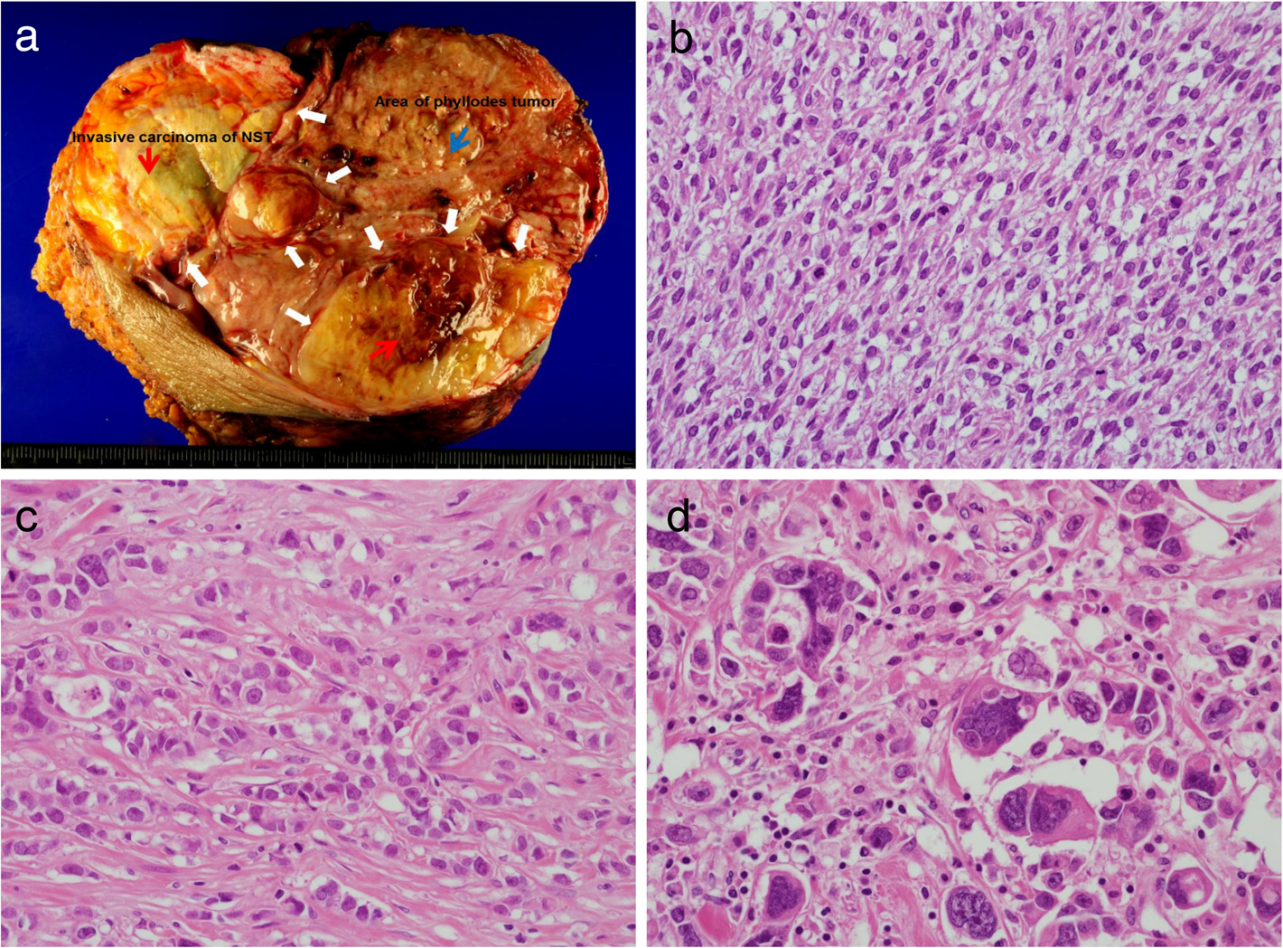
**Pathologic findings and photomicrographs of the rumor. (a)** Macroscopic examination of the left breast showed a well demarcated mass which composed of two separated tumorous lesions; phyllodes tumor (red arrow) and invasive carcinoma of no special type (blue arrow) with clearly defined margin (white thick arrows). **(b)** Malignant phyllodes tumor showing a marked stromal cell proliferation with brisk mitotic activity, resembling fibrosarcoma (original magnification × 400). **(c)** Area of invasive carcinoma of no special type (original magnification × 400). **(d)** Area with pleomorphic carcinoma (original magnification × 400).

**Figure 4:**
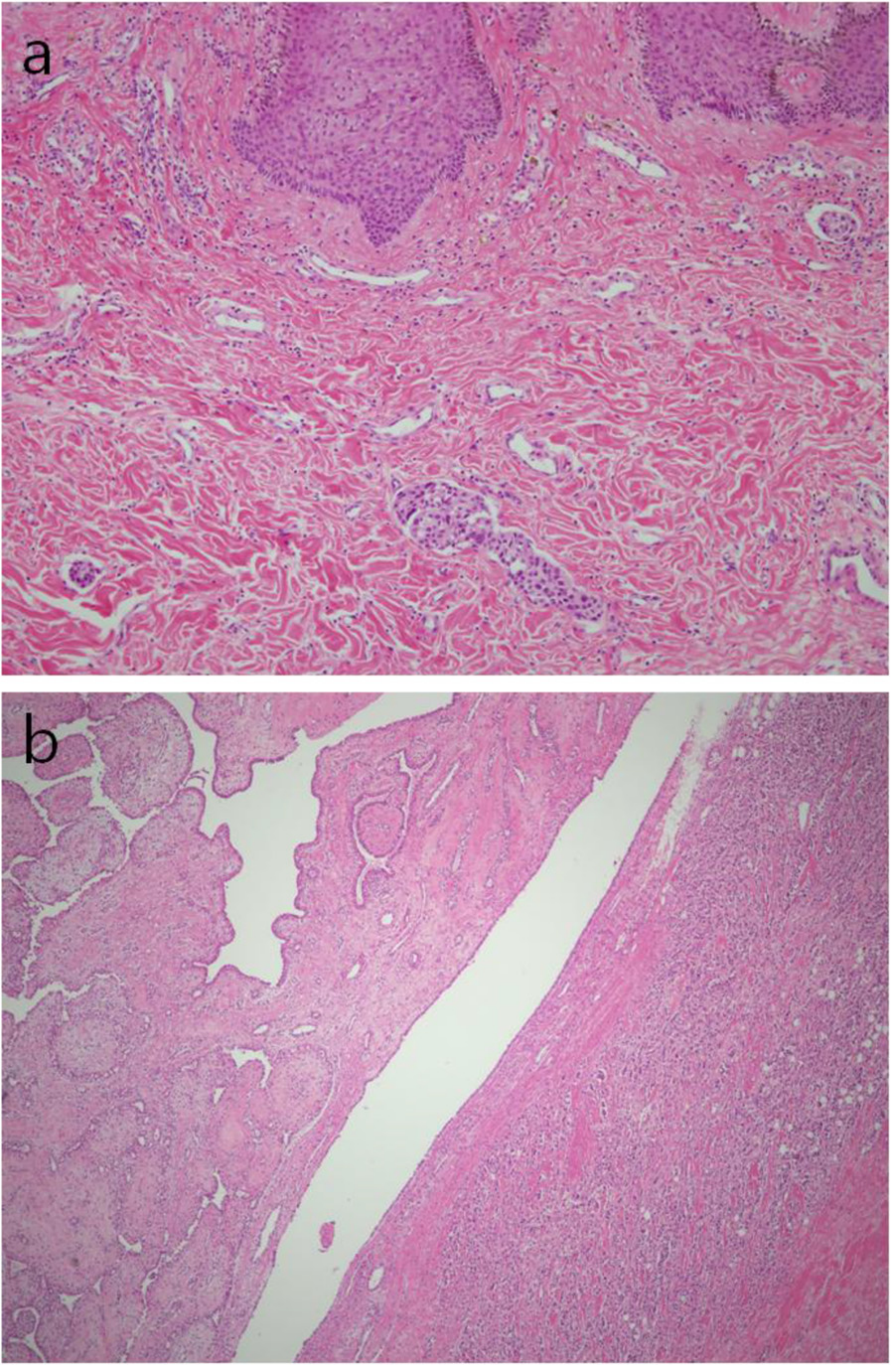
**Photomicrographs of the tumor. (a)** Thickened skin showing dermal lymphatic tumor emboli (original magnification × 100). **(b)** Collision tumor composed of phyllodes tumor and invasive carcinoma of no special type (original magnification × 40).

Under strong clinical suspicion of inflammatory breast cancer, we performed a core needle biopsy on the tumor itself. Core-cut biopsy revealed a biphasic neoplasm with mild nuclear atypia and low mitotic activity, accompanied by leaf-like processes protruding into dilated ductal spaces, consistent with a low-grade phyllodes tumor. We discussed the discrepancy about the clinical diagnosis and core biopsy results at the preoperative multidisciplinary meeting that included an oncologist, a breast surgeon, a pathologist and a radiologist. We concluded the case was a carcinoma associated with huge phyllodes tumor and decided not to do further preoperative biopsy. The patient underwent a palliative modified radical mastectomy with level II axillary LN dissection. Macroscopic examination of the resection specimen revealed a firm and well-demarcated mass with hemorrhage and necrosis, measuring approximately 24 cm in the largest dimension (Figure 3a). This mass was composed of two separated tumorous lesions: phyllodes tumor and invasive carcinoma of no special type. Histologically, about 70% of the tumor area represented a classical phyllodes tumor showing variable benign to malignant histologic characteristics. The malignant phyllodes tumor was composed of highly cellular stromal cells, moderate and variable nuclear atypia, and increased mitotic activity (>10 mitotic figures per 10 high-power fields) (Figure 3b). In addition, in almost one-third of the tumor, frankly invasive carcinoma of no special type (Figure 3c) with foci of pleomorphic carcinoma was observed (Figure 3d). The skin was thickened and showed pathognomonic dermal lymphatic tumor emboli, consistent with inflammatory carcinoma (Figure 4a). The areas of margin between the two tumor lesions were evaluated by further re-cutting and serial section. On serial section, there was no histologic evidence of transition from phyllodes tumor to invasive ductal carcinoma, so the lesion was thought to be a collision tumor with invasive carcinoma of no special type and malignant PT, rather than ductal carcinoma arising from PT (Figure 4b). Metastatic ductal adenocarcinoma cells were detected in 16 out of 16 dissected axillary lymph nodes. On immunohistochemistry, both epithelial and stromal compartment of the PT as well as the carcinoma cells showed no immunoreactivity for estrogen and progesterone receptors and HER2, except for p53 overexpression on the carcinoma cells.

The wound healed completely without complication. Chemotherapy based on anthracyclines and taxanes was started and radiotherapy was planned. Six months after the operation, and after completion of eight cycle’s chemotherapy, the patient developed left supraclavicular and upper chest wall swelling, which was consistent with regional aggravation of metastatic carcinoma and was supported by image findings. She underwent a session of radiation therapy to the chest wall and neck, and platinum-based chemotherapy will be pursued in this patient as further treatment. She is doing well with good compliance to the chemotherapy and radiation therapy.

## Discussion

Although there have been some cases reported of coincidental presentation of breast carcinoma and PT in same breast, most of the cases were the carcinoma that arisesfrom the epithelial component of a malignant or benign PT [5-7]. On review of the literature, we found three cases of true collision tumor with malignant PT and invasive ductal carcinoma in the same breast (Table 1). In case 1 and case 2, collision tumors were composed of malignant PT with sarcomatous differentiation and ductal carcinoma in a separate manner [8,9]. But case 3 was a collision tumor of ductal carcinoma within malignant PT without transition. In case 3, a clonal examination was performed to differentiate the tumor and an analysis of the polymorphic microsatellite markers revealed a divergent loss of heterozygosity(LOH) pattern between stromal and epithelial components of the PT and the carcinoma, thereby representing a true collision tumor [10]. Similarly, our present case showed distinct presentation of the two separate tumors in same site without any interminglement. In addition, the spindle cell lesion reveals strong expression for vimentin and negative immunoreactivity for Pan-CK, CK5/6, HM-CK, and the tumor cells in the axillary LNs and skin lesions were compatible with those of invasive ductal carcinoma. So, the lesion was identified as a collision tumor with invasive ductal carcinoma and malignant PT, rather than as a type of carcinosarcoma.

**Table 1:** Summary of reported cases of collision tumor with breast phyllodes tumor and invasive ductal carcinoma

**No.**	**Author [Reference]**	**Year**	**Age**	**PT**			**IDC**			**Site**	**Surgery**	**Treatment**	**Outcome**	**F/U period (mo.)**
				**Type**	**Size (cm)**	**LNI**	**T stage**	**Size (cm)**	**N stage**					
1	L. Auerbach [8]	2002	69	M		(-)	T1b		N1	Separate site	PM AD	ET (TAM)	LR of PT in 40 mo.	51.
													Died of lung metastasis of PT	
2	Kefeli M *et al*. [9]	2008	26	M	4.5	(-)	T2	2.5	N1 (1/22)	Separate site	MRM	CT RT	Died in 1 year	12
3	Macher-Goeppinger S *et al*. [10]	2010	70	M	6	(-)	T2	2.5	N0	Same site	MRM	CT RT		F/u loss
4^a^	Shin YD *et al*.	2013	45	M	24	(-)	T4d	8	N3c (16/16)	Same site	MRM	CT RT	Local recurrence of IDC in 6 mo.	14

^a^Present case.

AD, axillary dissection; CT, chemotherapy; ET, endocrine therapy; F/U, follow up; IDC, invasive ductal carcinoma; LNI, lymph node involvement; LR, local recurrence; M, malignant; MRM, modified radical mastectomy; PM, partial mastectomy; PT, phyllodes tumor; RT, radiation therapy; TAM, tamoxifen.

Radiologically, magnetic resonance image (MRI) can be a useful tool for the diagnosis of breast PT. PTs showed internal non-enhanced septations, silt-like patterns in enhanced images and signal changes from T2-weighted to enhanced images correlated with the histologic grade [11,12]. MRI showed findings typical of ductal carcinoma, an irregular mass with segmental, regional enhancement demonstrating a rapid increase in signal intensity following contrast enhancement followed by rapid washout. A preoperative MRI could have elevated the diagnostic accuracy in the present case, but we would have been unable to study an MRI because of the huge mass. Also, although there were some differences in metabolism in PET/CT image between PT and ductal carcinoma, they were not pathognomonic. Unfortunately we could not distinguish PT and ductal carcinoma in the preoperative CT and PET/CT images because there was a wide area of necrosis in both the huge PT and in the inflammatory breast carcinoma.

The clinical prognosis of collision tumor may be influenced by the subtype and pathologic stage of the more aggressive tumor in the breast. The prognosis for cases consisting of carcinoma and benign PT may be favorable, however, when a carcinoma lesion is detected at an early stage or as an *in situ* lesion due to a rapidly growing PT [6,7]. In our case, invasive carcinoma of no special type may be considered to have a greater adverse effect on clinical prognosis than the PT due to extensive dermal lymphatic tumor emboli and metastatic ductal carcinoma in the axillary lymph nodes. Furthermore, invasive carcinoma showed triple negative breast cancer (TNBC) with neither expressed hormonal receptors noroverexpressed HER2. Chemotherapy is the only systemic treatment available for TNBC. Cytotoxic therapies, anthracyclines or taxanes, achieved good tumor regression rates in the neo-adjuvant setting, but also showed considerable recurrence. Platinum-containing regimens should be considered as an important treatment option for in both the neoadjuvant and metastatic setting [13]. Even though inflammatory breast carcinoma is often considered a systemic disease, the risk for locoregional recurrence remains an important clinical problem, particularly for patients with triple negative inflammatory breast carcinoma. Therefore, locoregional treatment intensification should be considered on an individual basis. After completion of anthracyclin- and taxane-based chemotherapy, our patient showed left upper chest wall and supraclavicular swelling, consistent with regional aggravation of metastatic carcinoma. After the completion of radiation therapy to the chest wall and neck, platinium-based chemotherapy is scheduled.

## Conclusions

We reported a rare case of a collision tumor presented as inflammatory breast carcinoma of a ductal adenocarcinoma and malignant PT. Lacking standardization, treatment of these extremely rare cases should therefore be tailored to treat PT and carcinoma individually. A multimodal approach, including an extensive surgery that includes axilla, may be warranted, as well as an adjuvant treatment with radiation therapy and chemotherapy.

## Consent

Written informed consent was obtained from the patient for publication of this Case report and any accompanying images. A copy of the written consent is available for review by the Editor-in-Chief of this journal.

## Abbreviations

CT: Computed tomography; LN: Lymph node; MRI: Magnetic resonance imaging; PET: Positron emission tomography; PT: Phyllodes tumors.

## Competing interests

The authors declare that they have no competing interests.

## Authors’ contributions

YJC made substantial contributions to conception and design, and acquisition of data. YDS and SKL drafted the manuscript and revised its final form. KSK performed radiologic interpretation and MJP, HSY, JHK performed pathohistological and immunohistochemistry evaluation. YDS and YJC gave final approval for version to be published. All authors read and approved the final manuscript.
